# 3D Static Point Cloud Registration by Estimating Temporal Human Pose at Multiview

**DOI:** 10.3390/s22031097

**Published:** 2022-01-31

**Authors:** Byung-Seo Park, Woosuk Kim, Jin-Kyum Kim, Eui Seok Hwang, Dong-Wook Kim, Young-Ho Seo

**Affiliations:** 1 Department of Electronic Materials Engeering, Kwangwoon University, Kwangwoon-ro 20, Nowon-gu, Seoul 01897, Korea; bspark@kw.ac.kr (B.-S.P.); kws@kw.ac.kr (W.K.); jkkim@kw.ac.kr (J.-K.K.); dwkim@kw.ac.kr (D.-W.K.); 2 Yeshcompany, 18, Teheran-ro 43-gil, Gangnam-gu, Seoul 06151, Korea; ushwang@yesh.co.kr

**Keywords:** point cloud, 3D registration, RGB-D, joint set, pose estimation

## Abstract

This paper proposes a new technique for performing 3D static-point cloud registration after calibrating a multi-view RGB-D camera using a 3D (dimensional) joint set. Consistent feature points are required to calibrate a multi-view camera, and accurate feature points are necessary to obtain high-accuracy calibration results. In general, a special tool, such as a chessboard, is used to calibrate a multi-view camera. However, this paper uses joints on a human skeleton as feature points for calibrating a multi-view camera to perform calibration efficiently without special tools. We propose an RGB-D-based calibration algorithm that uses the joint coordinates of the 3D joint set obtained through pose estimation as feature points. Since human body information captured by the multi-view camera may be incomplete, a joint set predicted based on image information obtained through this may be incomplete. After efficiently integrating a plurality of incomplete joint sets into one joint set, multi-view cameras can be calibrated by using the combined joint set to obtain extrinsic matrices. To increase the accuracy of calibration, multiple joint sets are used for optimization through temporal iteration. We prove through experiments that it is possible to calibrate a multi-view camera using a large number of incomplete joint sets.

## 1. Introduction

Recently, RGB-D sensors (cameras) combining RGB and depth sensors have become common and are widely used in various fields. The RGB-D camera helps to accurately and quickly extract the shape of an object and the 3D structure of the surrounding environment. RGB-D cameras have developed various fields such as SLAM and navigation [1,2], tracking [3,4], object recognition and localization [5], pose estimation [6], and 3D model registration [7]. The color components of the RGB-D camera are obtained using the RGB camera. On the other hand, depth information is obtained using various methods such as time-of-flight (ToF) cameras, laser range scanners, and structured-light (SL) sensors [8]. RGB-D cameras include the Azure Kinect of Microsoft [9], the Phoxi 3D of Photoneo [10], the Zivid Two of Zivid [11], the Helios of Lucid [12], and the RealSense of Intel [13]. These cameras have various properties (operational time, depth accuracy, cost, sensing method) according to their intended usage. Since human pose estimation is used for extrinsic calibration, the sensing method of using a laser is not suitable for this study, although it has a high degree of depth accuracy. The temporal calibration and registration for humans in motion require a high frame rate to capture and calculate depth map and RGB image, so a camera that uses a long operation time is not suitable for this study. For reliable and accurate scene representation using RGB-D cameras, intrinsic calibration of each camera and extrinsic calibration between two sensors are required. Recently, intrinsic parameter sets are being determined in advance, and these values are stored in non-volatile memory inside the device. In applications that perform imaging using multiple RGB-D cameras, such as real-time scanning/integration and capturing 3D geometry models, extrinsic calibration between multiple cameras is very important [14]. Since RGB-D cameras acquire both RGB and depth information, calibration between multi-view RBG-D cameras uses depth information, unlike the classical method such as multi-view camera-based calibration using only RGB information.The multi-view installation implies the existence of multiple cameras and simultaneous shooting. When performing calibration using RGB information and depth information simultaneously, the depth information generation method and process are considered [15]. To this end, we use a 3D human joint set (skeleton) as feature points to calibrate multi-view RGB-D cameras.

Various studies have been conducted to obtain accurate camera parameters. Methods can be classified into a structured light-based depth-sensing method [16,17,18], and a ToF camera-based calibration method for depth sensing [19,20,21,22]. Considering noise removal, pattern generation, sensor quality, depth error prediction and correction, and thermal and environmental distortion [23] for successful calibration, it is not easy to clearly define the scope of the calibration technique. For calibration, there are methods of using a chessboard and using feature points of an image without using a chessboard. However, once a multi-view camera system is installed, it is very cumbersome to recalibrate due to the physical movement of the camera. In using a chessboard, it is necessary to bring a 2D or 3D chess board and perform a calibration after capturing an image while moving. The method of finding feature points after photographing an object and performing calibration using the feature point has the advantage of not requiring a chessboard. Still, there is a difficulty in finding an exact and consistent feature point. Moreover, it has properties that change the result. The human pose has been considered a good candidate for the feature point. The various approaches to calibration based on the human pose estimation have been researched. Lee et al. proposed a robust registration method of multiple RGB-D cameras. They used a human-body tracking system with the Azure Kinect SDK to estimate a coarse global registration between cameras. To overcome global registration errors, they propose a registration refinement procedure for removing calibration mismatches [24]. Takahashi et al. proposed an algorithm for estimating 3D human poses from multi-view videos captured by unsynchronized and uncalibrated cameras by relaxing the reprojection errors to avoid optimizing to noised observations and introduce a geometric constraint on the prior knowledge that the reference points consist of human joints [25]. Yoon et al. studied a targetless method for calibrating the extrinsic parameters among multiple cameras and a LiDAR sensor for object pose estimation, which exploited any objects of unspecified shapes in the scene to estimate the calibration parameters in a single-scan configuration [26]. In these previous works, there was no attempt at temporal calibration and no application of 3D reconstruction with which to verify the numerical accuracy of extrinsic parameters in the application. This paper develops a new methodology for temporally calibrating multiple cameras, randomly located in space without a special calibration board, using joints as feature points [27], and then reconstructing point clouds captured from multiple cameras. Especially, we use incomplete skeletons for extrinsic calibration and enhance the extrinsic parameters by temporally updating them using gradient descent of the loss function.

This paper is organized as follows. First, Section 2 describes obtaining a camera transformation matrix based on an optimization function used for registration between joint sets. Then, Section 3 proposes a calibration algorithm. Section 4 shows the experimental results, and Section 5 concludes our thesis.

## 2. Multi-View Extrinsic Calibration Based on Human Pose

This section describes the multi-view camera system we use and how to obtain extrinsic parameters using a human pose.

### 2.1. Multi-View Camera System

**Figure 1:**
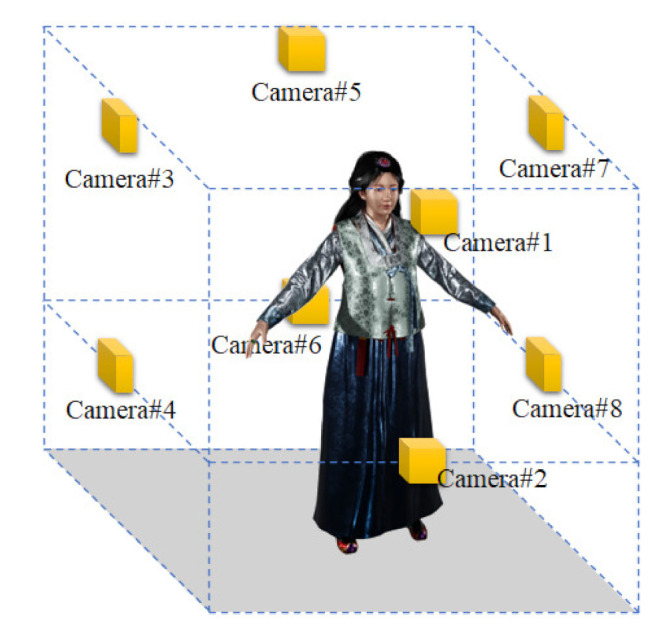
Distributed camera system for scanning photorealistic 3D volumetric model based on point cloud.

The multi-view camera system places several cameras at arbitrary positions in space and scans an object. To generate a 3D volumetric model, we install eight cameras in space. Eight cameras face the center of the space; four cameras below and four cameras above. Since extrinsic calibration is performed for multi-view cameras, the cameras do not need to be installed in precise locations. Figure 1 shows the layout of the multi-view camera system we use. The cameras are arranged in consideration of the type and performance of the RGB-D sensor and the size of an object to be scanned. The maximum scanning quality and the number of frames per second depend on the RGB-D sensor’s characteristics. We discuss the RGB-D sensor using Azure Kinect, a relatively low-cost ToF sensor [28].

### 2.2. Extrinsic Calibration

**Figure 2:**
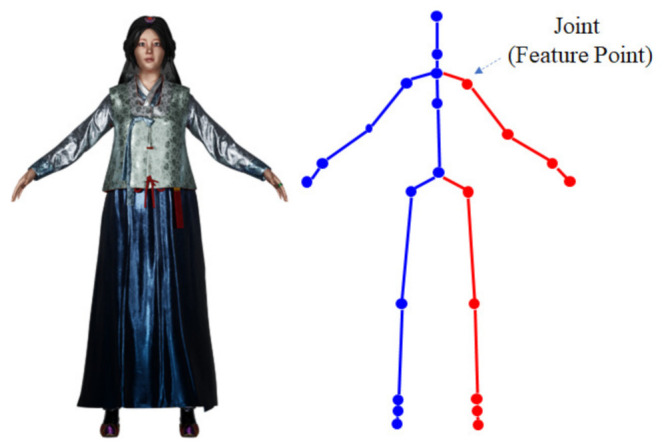
Joints as feature points in human pose estimation.

**Figure 3:**
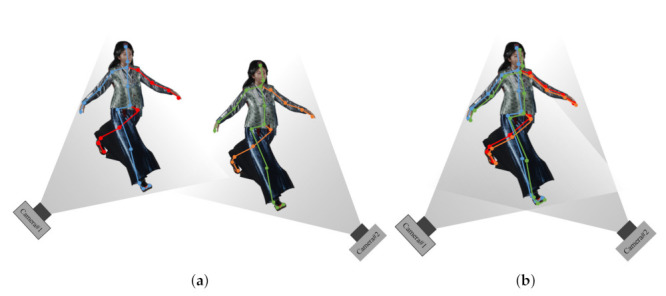
Initial parameter generation through joint set selection and primary joint alignment (**a**) before calibration, (**b**) after calibration.

First, a 3D human pose of Figure 2 is used to find a matching point in an RGB image input from multiple cameras. Figure 1 gives an example of a human, and Figure 3 is the result of displaying the joints for feature points [29]. Since the performance of a pose estimation based on deep learning is sensitive to the condition of the object, the feature point by the pose estimation may have lower accuracy than the case of the special board. In order to obtain 3D coordinates of the feature points, calibration between the depth and the RGB image is performed, and 3D coordinates of the matching points are obtained from the depth map [29].

Next, we use a method for obtaining extrinsic parameters of each camera using matching coordinates in point cloud sets for registration [30]. These parameters are calculated using an optimization algorithm such that the squared Euclidean distance (SED) of the matched coordinates is minimal. The transformation matrix of the coordinate system includes parameters for rotation angles and translation values for each of the *x*, *y*, and *z* axes. After setting one camera as the reference coordinate system, the parameters for converting those of other cameras to the reference coordinate system are obtained. $X_{ref}$ represents the coordinates of the reference camera and $X_{i}$ represents the coordinates of the remaining cameras. $R_{i\to ref}$ and $t_{i\to ref}$ represent the rotation and translation matrix from each camera to the reference camera. The initial $R_{i\to ref}$ is a unit matrix and $t_{i\to ref}$ is all zero. When Equation (1) is applied with the initial parameter, the result is $X_{i}$, and converges to $X_{ref}$ during optimization [31].

(1) $$X_{i}^{{}^{\prime }}=R_{i\to ref}X_{i}+t_{i\to ref}$$

The loss function to be optimized is the average value of SED of $X_{ref}$ and $X_{i}^{{}^{\prime }}$. Equation (2) represents the error function.

(2) $$f_{Error}=\frac{1}{N}\sum_{j=0}^{N}{∥X_{ref}\left(j\right)-X_{i}^{\prime }\left(j\right)∥}_{2}^{2}$$

The process of differentiating the loss function with respect to the coordinate transformation parameters and updating the parameter to minimize the function can be expressed as Equation (3). α is a learning rate constant, and a value of 0.01 was used. $P_{n+1}$ and $P_{n}$ are parameters in the *n*+1 and *n*-th iterations, respectively.

(3) $$P_{n+1}=P_{n}-\alpha \frac{\partial f_{Error}}{\partial P_{n}}$$

When the parameters of each camera are obtained after the convergence of Equation (3), the transformation from the camera coordinate system to the world coordinate system can be performed using Equation (4), and the point cloud can be aligned based on the unified coordinate system. represents world coordinates (reference camera coordinates), and represents camera coordinates [30,31].

(4) $$P_{C}=R\times P_{W}+t$$

## 3. Proposed 3D Static Reconstruction

In this section, we propose a graphics pipeline that can reconstruct a point-cloud-based 3D model using a 3D joint set in a multi-view camera system. Our system assumes that the cameras are fixed at some positions for all frames, and the human moves between the cameras.

### 3.1. Extrinsic Calibration

Figure 3 shows a conceptual diagram of a transformation matrix between cameras using a 3D joint set in a multi-view camera system environment. This paper uses an Azure Kinect as the RGB-D sensor, and the human pose is estimated using Azure Kinect’s SDK [32] and MediaPipe [33]. Joints acquired based on each camera coordinate system are not aligned in space. However, if the coordinate transformation matrix between the two cameras is obtained using the method described in Section 2, the result shown in Figure 3b can be obtained. In the process of matching the joint set predicted by the two cameras, a coordinate transformation matrix between the two cameras is obtained, and the two cameras can be aligned based on one common world coordinate system.

**Figure 4:**
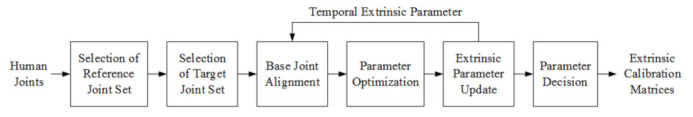
Proposed joint-based extrinsic calibration.

Next, the proposed extrinsic calibration will be described. Both methods can output a 3D joint set. However, it does not matter which pose estimation algorithm is used for each camera, and it does not matter if a different algorithm is used for each camera. Therefore, the joint set obtained for each camera may not have all the joints. If the estimated joint set has a subset of the entire joints, there is no problem performing calibration using only the acquired joints. The external calibration algorithm proposed by us is summarized using the flowchart in Figure 4.

**Figure 5:**
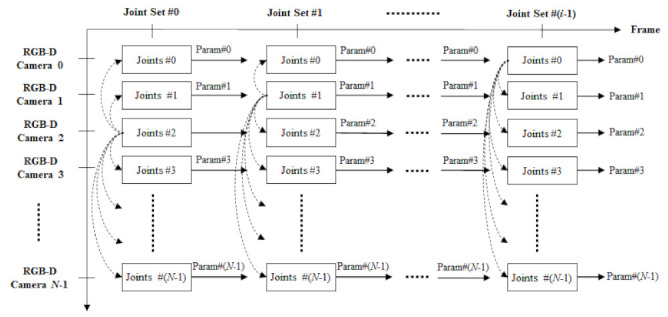
Temporal camera selection and parameter updating for the joint-based extrinsic calibration.

A reference joint set is first selected among the joint sets. Although the selection of the reference joint set does not affect the overall performance much, in general, one of the joint sets with the most joints is selected as the reference joint set. next, the target joint set is chosen in order that many joints overlap with the joints of the reference joint set. These two joint sets are first aligned with respect to their primary joints (vertebra and pelvis). The primary joints are defined as the two or three vertebra and pelvis joints. The used pose estimation algorithm decides the number of the joints because pose estimation algorithms may have different human skeletons. Next, based on the optimization function described in Section 2.2, the coordinate transformation parameters of the camera are obtained while matching two joint sets. This process is repeatedly performed for all joint sets, and this process is repeatedly performed for many frames until it converges to a constant error value. Finally, when the error converges, the parameter is determined as the final coordinate transformation parameter. The process of optimizing extrinsic parameters using multiple frames is explained using Figure 5.

In Figure 5, the proposed technique for multiple frames is visually expressed. Using a joint set in multiple frames, each camera’s external parameters (Param#*N*) are obtained. Each camera can capture only a part of the human depending on the location where it is installed and can estimate only some joints of the joint set. For this reason, the number of the estimated joints as the feature points for extrinsic calibration can be variable, so it does not matter that the estimated joints are incomplete for each human. If the number of the estimated joints is less than the minimally required feature points (four joints in this paper), the skeleton should not be used in extrinsic calibration. If the joint set of a certain camera does not satisfy the minimum requirement, the calibration for this camera can be temporally continued in the next frames until the extrinsic parameter of the corresponding camera is estimated. As explained earlier, the extrinsic calibration is performed with overlapping joints of multiple frames. Joint sets without primary joints are excluded from calibration, and if there are fewer than four overlapping joints, calibration is performed after selecting a new reference joint set. After the finally acquiring and selecting the external parameters (Param#*N*), 3D static registration is performed using the joint set information predicted from each camera.

Considering the case of two cameras, 3D human pose is estimated from two 3D sensors. The joints of the joint set obtained through this are regarded as feature points, and calibration is performed using 3D joint sets obtained from two 3D sensors. As for the calibration result, the 3D pose estimation used depends on the quality of the estimated 3D joint set. To alleviate this dependence, this process should be repeated over time. In addition, an algorithm that compensates for estimation failures that irregularly occur in 3D pose estimation results may be needed.

### 3.2. 3D Registration

**Figure 6:**
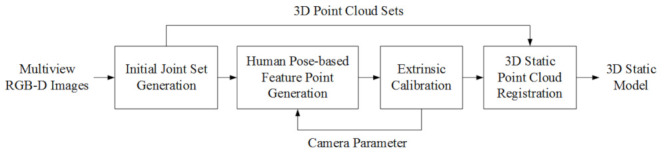
The proposed algorithm for 3D static registration.

This section describes the overall process for 3D registration. When the transformation parameters of the multi-view cameras are previously obtained, 3D registration may be automatically performed. As described above, to perform calibration using a joint set, we use several frame sets. First, joint sets for these multiple frame sets are estimated (initial joint set generation). Then, joints of joint sets estimated from multiple frames are selected as feature points (human pose-based feature-point generation). First, one frame set is selected from among several frame sets. In this frame set, a joint set obtained from one camera is selected as a reference joint set, and one of the remaining joint sets is selected as a target joint set. When selecting two joint sets, at least three or more joints, including the reference joint, should satisfy the corresponding condition. Next, the two selected joint sets are aligned based on the primary joint. Through this, transformation parameters between the two cameras are obtained. This process is repeatedly performed for several frames and is performed until the transformation parameters converge (extrinsic calibration). Since the unaligned multi-view camera system is distributed at an arbitrary location, all or parts of the object may be included in the image acquired by each camera according to the size or location of the object. In the worst case, all cameras may capture only a subset of objects. In the case of a multi-view camera, as the object moves, the part of the object that each camera can capture may vary. In this case, extrinsic calibration is performed only with partial information captured by each camera. When extrinsic calibration is completed, a 3D model is generated by integrating the 3D point cloud using the transformation matrix (3D static registration) [34]. In Figure 6, the 3D static reconstruction algorithm is depicted.

Through the proposed calibration process, all cameras except one at the reference position have optimized coordinate transformation parameters [$R_{i\to ref}*S_{i\to ref}$∣$t_{i\to ref}$], where $S_{i\to ref}$ is a scaling matrix. These extrinsic parameters are applied to the point cloud obtained from each camera to align with the reference coordinate system, which is a simple affine transformation. This process is defined as 3D registration. Equation (5) is a process of transforming the point cloud to the reference coordinate system by applying the coordinate transformation parameter [$R_{i\to ref}*S_{i\to ref}$∣$t_{i\to ref}$] to the point cloud generated by the *i*th camera. Here, $PC_{i}$ is the coordinates of the point cloud obtained from the *i*th camera, and $PC_{ref}$ is the coordinates of the point cloud transformed to the reference coordinate system. When all point clouds acquired by RGB-D cameras are aligned regarding the reference coordinate system, a 3D volumetric model by multi-view RGB-D cameras is generated. The result of transforming the coordinates using the extrinsic parameter corresponds to the 3D static registration result. This result is the same as the generated 3D model.

(5) $$PC_{ref}=\left[R_{i\to ref}*S_{i\to ref}|+t_{i\to ref}\right]PC_{i}$$

## 4. Experimental Result

This section describes the experimental results of the proposed calibration technique for human pose-based 3D static registration. First, the experimental environment is introduced, and then the numerical results of the calibration for each camera are shown. Finally, the effectiveness of the proposed method is demonstrated by showing the 3D registration results generated using the camera matrix obtained through calibration.

### 4.1. Environment

**Figure 7:**
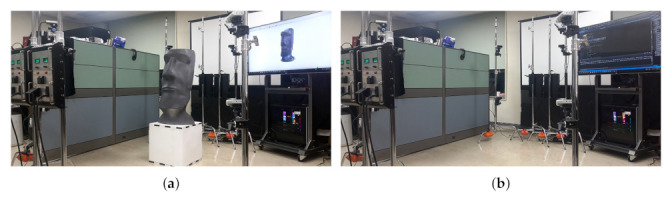
Experimental environment (**a**) capturing, (**b**) camera system.

Figure 7 shows a picture of the experimental environment. For the experiment, eight Azure Kinects with a total of eight ToF (time of flight) RGB-D (depth) sensors were used. The camera can be located in various positions, and we experimented by placing two cameras on four sides. Eight cameras are input to one workstation through an optical cable-type USB 3.0 interface. Our system could operate the 3D registration in real-time (30 frames per second).

### 4.2. 3D Pose Estimation Result

**Figure 8:**
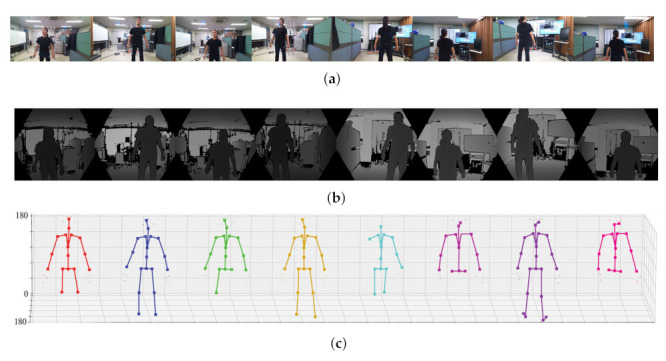
3D pose estimation result using the SDK of Azure Kinect (**a**) RGB image, (**b**) calibrated depth image, (**c**) estimated joint set in eight view-points.

**Figure 9:**
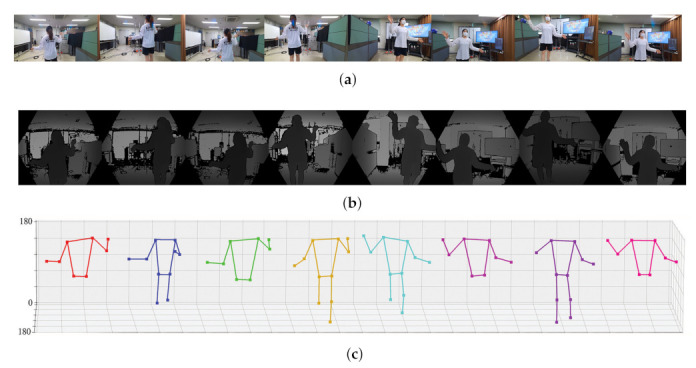
3D pose estimation result using the deep learning of MediaPipe (**a**) RGB image, (**b**) calibrated depth image, (**c**) estimated joint set in eight view-points.

The resulting images captured by each camera and the extracted joint set are summarized in Figure 8 and Figure 9. We estimated human poses for two humans using two different methods (SDK of Azure Kinect, MediaPipe). Figure 8 is the result of human pose estimation using the SDK provided by Azure Kinect, and Figure 9 is the estimation result using the deep learning solution provided by MediaPipe. Both methods can estimate 3D pose using depth in common. In the two figures, (a) is the RGB image output from eight cameras, (b) is the depth images, and (c) is the result estimated using (a) and (b). Since the two methods express the joint set differently, Figure 8c and Figure 9c show different shapes. An important point to observe in Figure 8c and Figure 9c is that each camera cannot capture the entire object (person). According to the relative position of the camera and the object (person), each camera can only capture a part of the person and can estimate only the joint set of part of the person. The proposed method performs calibration using the joint of an incomplete joint set with only partial information as feature points.

### 4.3. Extrinsic Calibration Result

**Figure 10:**
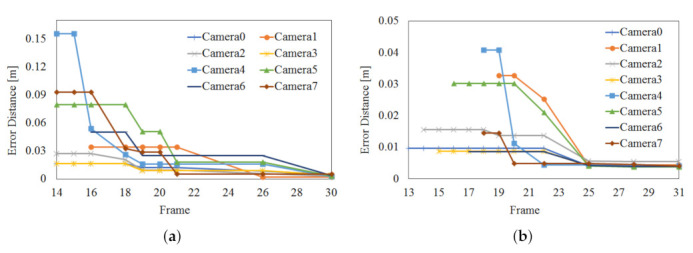
Optimization result of camera parameter based on joints of human 3D joint set (**a**) SDK of Azure Kinect, (**b**) deep learning model of MediaPipe.

Figure 10 shows the optimization process of camera parameters using the joints of the joint set obtained from 8 viewpoints. After the optimization is completed through the process shown in Figure 10, 3D registration is possible in real-time using the 3D point cloud. Considering the optimization process of viewpoint 1 (reference) and viewpoint 2 (target), a transformation matrix is a obtained so that the overlapping joints of viewpoint 1 and viewpoint 2 can be overlapped in 3D coordinates in space. That is, an optimized matrix that can move the joint of viewpoint 2 to viewpoint 1 is obtained. The proposed algorithm performs optimization to obtain camera parameters for multiple frames. The experiment was conducted to stop the optimization when the error for the transformation result between the two viewpoints by the camera matrix obtained through optimization convergence to a constant value. In the case of the Azure Kinect in Figure 10a, optimization was carried out over about 30 frames (about 1 min). The position error by the transformation matrix finally converges to an average of 3.48 mm. In the case of Azure Kinect in Figure 10b, optimization progressed over about 74 frames (about 2.5 min). Finally, the position error by the transformation matrix converged to an average of 4.42 mm. This experiment confirmed that a transformation matrix with an error of about 3 to 4 mm could be obtained regardless of the type of 3D human pose estimation algorithm. In Figure 10, the frame numbers corresponding to the *x*-axis represent frames for significant updates.

**Figure 11:**
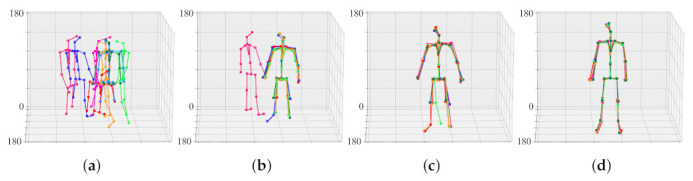
Progress of calibration and 3D joint set estimation according to frame. Each raw corresponds to a view-point of a 3D model. (**a**) initial joint sets, (**b**) joint sets in the 15th frame, (**c**) joint sets in the 21st frame, (**d**) joint sets in the 30th frame.

Before calibration, joint sets generated from eight viewpoints are each scattered in space. This is because the cameras are not calibrated. By merging eight joint sets into one joint set, the cameras are aligned in a common coordinate system. That is, the joint set aligns the camera, and the camera aligns the joint set again. Figure 11 shows the result of aligning the objects of Figure 8 through the calibration process. When the 30th frame is reached, the positions of the joint sets converge to almost the same three-dimensional coordinates. That is, the camera transformation matrix in the 30th frame is practically optimized.

**Figure 12:**
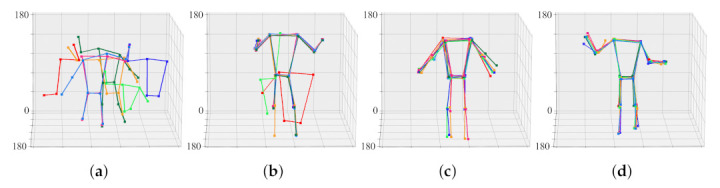
Progress of calibration and 3D joint set estimation according to frame. Each raw corresponds to a view-point of a 3D model. (**a**) initial joint sets, (**b**) joint sets in the 18th frame, (**c**) joint sets in the 73rd frame, (**d**) joint sets in the 74th frame.

Figure 12 shows the result of aligning the objects of Figure 9 through the calibration process. When the 74th frame is reached, the positions of the joint sets converge to almost the same three-dimensional coordinates. That is, the camera transformation matrix in the 74th frame is practically optimized. For ease of calibration and optimization, the face, fingers, and feet joints were excluded from the calculation.

### 4.4. Extrinsic Calibration Result

**Figure 13:**
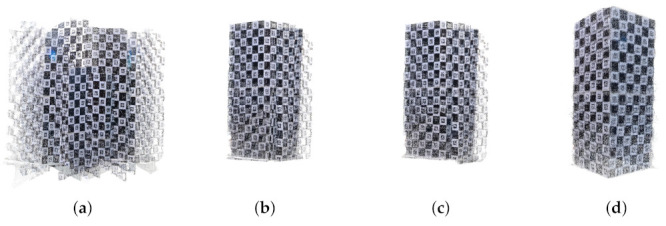
Calibration and registration result of the Charuco box (**a**) before calibration, (**b**) after 15 frames, (**c**) after 21 frames, (**d**) 30 frames.

This section describes the 3D registration results after multi-view extrinsic calibration. 3D registration was performed using the Charuco board, for which we already know the size and all 3D information. Figure 13 shows the 3D registration results using the 3D Charuco board. Figure 13a is the result before calibration, and Figure 13b–d is the 3D registration results using the camera transformation matrix by extrinsic calibration at frames 15, 21, and 30, respectively. As to the optimization progresses, the error of the camera transformation matrix is decreased, and the quality of the 3D registration is continuously improved. In Figure 13a, the point clouds by each camera were not aligned, but in Figure 13b, the point clouds were well-aligned, and the 3D Charuco board model was well registered.

**Figure 14:**
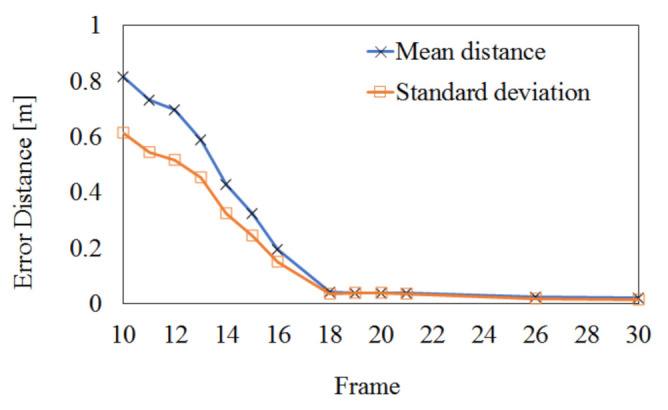
Numerical result of 3D registration error of the Charuco board.

In order to quantitatively evaluate the effect of the proposed extrinsic calibration on the 3D registration performance, Figure 14 shows the registration error of the Charuco board by the proposed algorithm. The registration error is estimated by the Euclidean distance between the ground truth (the physically measured or computer-generated model) and the registered model. First, we located two models in the 3D virtual space and calculated the 3D point-to-point distance corresponding to the error (or difference) between the two models. Then, we repeated locating two models and calculating distance until finding the minimum mean distance. The 3D point-to-point distance is defined as the distance between the nearest points of the two models. In Figure 14, the initial mean error of 0.816 m is reduced to 0.0203 m after 30 frames and the initial standard deviation of 0.613 m is reduced to 0.0163 m. Since this experiment selected 1024 feature points existing in the Charuco board, it may have very high accuracy.

**Figure 15:**
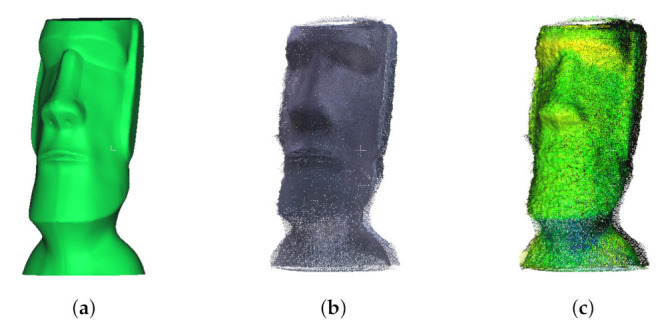
Calibration and registration result of the Moai statue (**a**) original 3D model, (**b**) after calibration, (**c**) superimposition of (**a**,**b**).

Figure 15 shows the results of the 3D registration of the Moai statue. We printed the Moai statue in Figure 15c with a 3D printer and used it in the experiment. In other words, we know all the 3D information of the actual Moai statue. It is rather difficult to judge the results of the three-dimensional point cloud as a two-dimensional image. However, it can be seen that the result of Figure 15c is very similar to the original object of Figure 15a. Figure 15c has the mean distance of 2.284 cm and the standard deviation of 4.036 cm. Therefore, a 3D object could be expressed with a relatively accurate 3D graphics model using the proposed method.

**Figure 16:**
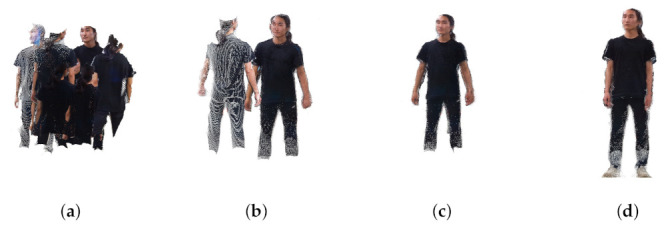
Generated point cloud (human #1) (**a**) before calibration, (**b**) after 15 frames, (**c**) after 21 frames, (**d**) 30 frames.

**Figure 17:**
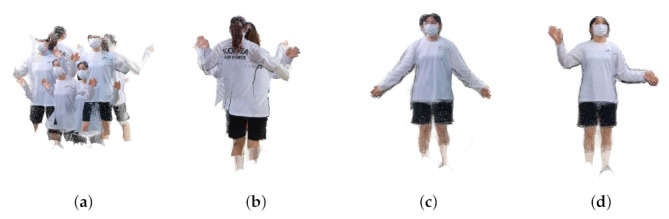
Generated point cloud (human #2) (**a**) before calibration, (**b**) after 18 frames, (**c**) after 73 frames, (**d**) 74 frames.

Figure 16 and Figure 17 show the results of 3D point cloud generation using the proposed technique. Figure 16a is the point cloud taken using eight RGB-D sensors. Figure 16b is the point cloud result obtained by 3D registration using the transformation matrices obtained after optimization for 15 frames. Figure 16b,c show the 3D registration results after 21 frames and 30 frames, respectively. In the case of Figure 17, the optimization result was converged upon after more frames were required. When comparing Figure 16 and Figure 17, although the number of frames required for optimization is different, the 3D registration results are visually similar in quality.

## 5. Conclusions

In this paper, when multiple cameras are located in space, we propose an algorithm that automatically calibrates multi-view cameras and performs 3D registration when a person is present. In other words, it uses the fact that the human pose can be estimated relatively consistently. In the process of matching the positions of the joint sets obtained from each camera, a camera transformation matrix between the cameras was obtained. Through this, all cameras could be positioned in a common-world coordinate system, and a 3D model could be expressed using a 3D point cloud. Using the pose estimation based on deep learning may increase the complexity of the extrinsic calibration and decrease the accuracy of the feature points by the dependency for the surficial condition of an object. The process of finding the relationship between the cameras used an optimization function, and as a result, the proposed calibration had an error of about 3 cm to 4 cm. We measured quantitative accuracy by experimenting on two objects for which we know the correct information. In the case of the 3D charcoal board, the mean and standard deviation of the registration error by calibration could be lowered to about 2.03 cm and 1.63 cm. In the case of the Moai statue, it was confirmed that the mean and standard deviation could be reduced to about 2.2849 cm and 4.0363 cm or less, respectively. Finally, it was verified that a relatively accurate 3D point cloud could be generated through an experiment on a photorealistic person. Therefore, we have shown that the presence of a person can successfully generate a 3D point cloud without the use of a special chess board or Charuco board.
